# Changes in Referral Rates After the Mandate of Charging Additional Fees for Non-referral First Visits: A Controlled Interrupted Time-series Analysis

**DOI:** 10.2188/jea.JE20250285

**Published:** 2026-05-05

**Authors:** Arisa Iba, Takehiro Sugiyama, Yuta Taniguchi, Ai Suzuki, Taeko Watanabe, Hiroyasu Iso, Nanako Tamiya

**Affiliations:** 1 Institute for Global Health Policy Research, Bureau of Global Health Cooperation, Japan Institute for Health Security, Tokyo, Japan; 2 Department of Health Services Research, Institute of Medicine, University of Tsukuba, Ibaraki, Japan; 3 Diabetes and Metabolism Information Center, National Institute of Global Health and Medicine, Japan Institute for Health Security, Tokyo, Japan; 4 Graduate School, University of Tsukuba, Ibaraki, Japan; 5 Health Department, Tsukuba City, Ibaraki, Japan; 6 Health Services Research and Development Center, University of Tsukuba, Ibaraki, Japan

**Keywords:** cost-sharing, referral, interrupted time-series analysis, Japan

## Abstract

**Background:**

In April 2016, Japan mandated higher-level hospitals (ie, Special Functioning Hospitals [SFHs] and Regional Medical Care Support Hospitals [RMCSHs] with ≥500 beds) to charge additional fees for non-referral first visits to facilitate hospital function differentiation. The mandate expanded to RMCSHs with 400–499 beds and 200–399 beds in April 2018 and April 2020, respectively. We investigated changes in referral rates (proportion of referred to first-visit patients) before and after the fee’s implementation.

**Methods:**

Using a community-based insurance claims database from a single prefecture in Japan, we extracted claims for first visits to hospitals with ≥200 beds between April 2014 and March 2022 and calculated monthly referral rates to five hospital groups (SFHs, RMCSHs with ≥500, 400–499, and 200–399 beds, and non-designated hospitals with ≥200 beds). We conducted a controlled interrupted time-series analysis by hospital category, treating non-designated hospitals as controls.

**Results:**

Of 405,087 first-visit patients (mean age 54.9; standard deviation, 20.2 years; 53.2% female), 157,734 (38.9%) had a referral. The average referral rate to SFHs was high pre-mandate and did not increase. With the mandate, referral rates to RMSCHs with ≥500 beds and 400–499 beds rose by 5.10% points (95% confidence interval [CI], 1.84–8.35) in 2016 and 4.49% points (95% CI, 0.28–8.70) in 2018, respectively, and stabilized afterward. Referral rates to RMCSHs with 200–399 beds remained unchanged.

**Conclusion:**

Average referral rates increased when the additional fee was mandated for RMCSHs with ≥400 beds, although the influence on health outcomes remains unclear.

## INTRODUCTION

Healthcare systems in developed countries face increased utilization and expenditure due to rapidly aging societies.^1^ To maintain the sustainability of the system, the appropriate allocation of healthcare resources and savings in medical expenditure are urgent issues.

Many healthcare systems have introduced gatekeeping systems to differentiate between primary and specialty care. Gatekeeping is used both in tax-funded health systems, such as those in the United Kingdom and Spain, and in social health insurance systems, such as those in Switzerland and the Netherlands.^2^ Gatekeeping is associated with better quality of care,^3^ lower utilization of health services, and lower expenditures.^2^ The gatekeeping system usually requires patients to obtain a referral from a general practitioner (GP) to access a specialist^4^; some medical systems introduce financial incentives for the patient as voluntary gatekeeping, such as different coinsurance rates when visiting GPs without a contract in France^5^ or different copayments between GPs and specialists in Belgium,^6^ with the expectation of reducing unnecessary specialist consultations.^7^ Higher cost-sharing is associated with lower healthcare utilization. The RAND Health Insurance Experiment, a landmark randomized controlled trial that evaluated the effect of different coinsurance rates on medical service utilization in adults aged ≤62 years, revealed that a higher coinsurance rate reduced the number of outpatient visits and outpatient service expenditures.^8^^,^^9^ A study using a natural experimental approach, difference-in-differences, reported that an increase in physician office visit copayments reduced physician office visits in the older population.^10^ The price elasticities for outpatient visits ranged from −0.08 to −0.18 in adults^10^^–^^12^ and −0.13 to −0.19 in children.^13^

In Japan, patients are free to choose any level of healthcare facility regardless of disease severity and their insurance status.^14^ Many patients visit hospitals offering higher levels of care directly without referral because they believe these hospitals provide highly specialized medical care.^15^ Since the Ministry of Health, Labour and Welfare intended to address the functional differentiation and cooperation of outpatient medical care,^16^ the Ministry introduced an additional fee for the first visit without a referral (hereafter referred to as additional fee), which was charged in addition to coinsurance outside of insurance coverage, in 1996. The Ministry mandated that higher-level hospitals charge an additional fee (at least 5,000 Japanese yen) in April 2016 and expanded the number of hospitals subject to the mandate every 2 years (eFigure 1). This policy produced a difference in patient expenditure on the first visit without a referral by hospital function and scale: patients pay the first consultation fee (10–30% of 2,880 Japanese yen, depending on their coinsurance rate) only when they visit a clinic or small hospital; they must pay an additional fee along with the first consultation fee when they visit a second or tertiary care hospital without a referral. The policy was intended to increase the gatekeeping role of primary care and differentiate the functions of clinics and hospitals; however, changes in medical utilization since the introduction of additional fees have not been evaluated.

Therefore, we aimed to investigate changes in the referral rates of first outpatient visits before and after the introduction of the additional fee using community-based health insurance claims data in a prefecture in Japan. We also calculated the price elasticity of the first visit to a high-level hospital.

## METHODS

### Study design

This retrospective observational study used claims data collected regularly between April 2014 and March 2022 from the National Health Insurance (NHI) program managed by the Ibaraki Prefecture. The Japanese universal health coverage includes an employment-based insurance program, a community-based insurance program (known as NHI), and a late-elderly health insurance program.^17^ There is a uniform national fee schedule; however, coinsurance rates range from 10% to 30%, depending on the patient’s age. Each municipality manages the NHI program and covers citizens ≤75 years, including the self-employed, farmers, part-time workers, the unemployed, and their dependents.^17^ The NHI program covers approximately 40% of Japan’s total population, and one-third of its beneficiaries are aged 65–74 years. The population of Ibaraki prefecture was approximately 2.9 million, of which 0.9 million (30%) were covered under the NHI program in fiscal year (FY) 2014.^18^ The number of beneficiaries subsequently declined to 0.65 million (23%) in FY 2021.^19^ The area of Ibaraki Prefecture is approximately 6,000 km^2^; the number of clinics and hospitals per 100,000 population was 48.5 and 5.9, respectively, lower than the national average (70.1 and 6.4, respectively); and 24 out of 169 hospitals (14.2%) have ≥200 general beds.^20^

The Ministry of Health, Labour and Welfare certifies hospitals (mainly university hospitals) that have the capability of providing, developing, evaluating, and conducting training in advanced medical care as Special Functioning Hospitals (SFHs).^21^ Additionally, the Ministry approves hospitals that are sufficiently competent to provide regional medical care and support family doctors who play key roles in delivering such care as Regional Medical Care Support Hospitals (RMCSHs).^22^ Although these hospitals are expected to primarily treat patients upon referral, Japanese citizens can visit any healthcare facility without requiring referral letters.^14^ Since 1996, the Ministry has allowed hospitals to charge additional fees to reduce direct visits to higher-level hospitals. To further promote functional differentiation among hospitals, the Ministry mandated the imposition of additional fees for SFHs and RMCSHs with ≥500 beds from April 2016 (intervention 1),^23^ for RMCSHs with 400–499 beds from April 2018 (intervention 2),^24^ and for RMCSHs with 200–399 beds from April 2020 (intervention 3) (eFigure 1).^25^

This study was approved by the Institutional Review Board of our institute (approval no: 1845-2). The need for informed consent was waived due to the anonymized retrospective study design.

### Data sources and participants

We obtained enrolment and monthly claims data in an anonymized format under a research agreement between the prefecture and University of Tsukuba. Enrolment data included birth year and month, sex, and enrolment and withdrawal dates, and claims data included monthly individual-level information on outpatient and inpatient service use, expenditure, and hospital codes. We obtained information on hospital bed counts, hospital function, starting dates, and additional fee amounts as of July 1, 2023, from the website of the Regional Bureau of Health and Welfare (https://kouseikyoku.mhlw.go.jp/kantoshinetsu/index.html) and checked the changes in the additional fee amounts on each hospital’s website.

We first extracted all outpatient claims for the first consultation at hospitals with ≥200 beds between April 2014 and March 2022. We defined first-visit patients as those with claims for the first consultation, excluding those who had concomitant claims for emergency department visits and emergency transport. Patients are usually advised to visit the referred hospitals within 1–2 months, as their conditions may change over time. For example, some SFHs in other prefectures require patients to bring a referral letter written within approximately 1 month, as the condition could worsen. The number of participants and referral rates did not differ when we extracted first-visit patients with claims of referral documents charged within 30 and 60 days before the first consultation. Extending the extraction period increases the possibility of mistakenly linking referral letters to other medical institutions. Therefore, we defined referral patients as first-visit patients with claims for referral documents charged at different hospitals within 30 days before the first consultation. Details of the billing codes used in the extraction are provided in eTable 1. The age at the beginning of the month of the first visit was calculated based on birth year, month, and visit date. We excluded two medical complexes with adjacent clinics, as well as four hospitals that obtained approval as RMCSHs after the intervention. We obtained the number of hospital beds as of FY 2014^26^ and confirmed that the hospital categories had not changed at the included hospitals.

### Outcome

Our primary outcome of interest was the monthly referral rate of each hospital, calculated by dividing the number of first-visit patients with a referral by the total number of first-visit patients. Age adjustment was performed using the 2015 Japanese standard population.

### Defined study period

We considered the following four defined periods in our study:

- Period 1 (April 1, 2014, to March 31, 2016 [ie, FY2014, 2015]): The period before the additional fee was mandated.
- Period 2 (April 1, 2016, to March 31, 2018 [ie, FY2016, 2017]): The period when the additional fee was mandated in the SFHs and the RMCSHs with ≥500 beds.
- Period 3 (April 1, 2018, to March 31, 2020 [ie, FY2018, 2019]): The period when the additional fee mandate was expanded to RMCSHs with 400–499 beds.
- Period 4 (April 1, 2020, to March 31, 2022 [ie, FY2020, 2021]): The period when the additional fee mandate was expanded to RMCSHs with 200–399 beds.

### Statistical analysis

We compared the demographics of first-visit patients who received a referral with those who did not. We then calculated the average of crude monthly referral rates before and after an additional fee was introduced by categorizing hospitals into five groups: SFHs, RMCSHs with ≥500 beds, RMCSHs with 400–499 beds, RMCSHs with 200–399 beds, and non-designated hospitals with ≥200 beds.

We investigated the changes in referral rates before and after the additional fee was mandated using a controlled interrupted time-series analysis (ITSA) for each hospital category, treating non-designated hospitals with ≥200 beds as a control. An ITSA allows us to assess how much an intervention changed an outcome of interest immediately after the intervention and thereafter.^27^ It is suited to evaluate interventions introduced at a population level over a defined time period.^28^ The intervention was conducted at three time points: April 2016, April 2018, and April 2020. Our proposed impact model hypothesized a significant change in the monthly referral rate at the time that the additional fee was mandated in the corresponding hospital category and that a higher referral rate would be maintained. We employed an ordinary least squares model with robust standard errors, as referral rates (the proportion of first-visit patients who received a referral) are normally distributed. To account for seasonality, we incorporated Fourier terms (one fundamental and one harmonic pair of sine and cosine functions) into our model. We then calculated the price elasticities in each hospital category using the following equation^10^^,^^11^^,^^29^:

$$\text{Price elasticity}=\frac{Q_{2}-Q_{1}}{(Q_{1}+Q_{2})/2}÷\frac{P_{2}-P_{1}}{(P_{1}+P_{2})/2}$$

where Q_1_ and Q_2_ denote demand (referral rates in this study) before and after the intervention, respectively, and P_1_ and P_2_ denote prices before and after the intervention, respectively. Price elasticity was measured as the percentage change in demand associated with a 1% increase in the price of a service, indicating how consumers respond to price fluctuations. We assumed that the out-of-pocket payment for the first consultation was 850 yen (30% of 2,880 yen, assuming a 30% coinsurance rate, because 30% is the usual coinsurance rate in Japan except for those under 3 years or most of those ≥70 years), and the additional fee was 5,000 yen (the minimum required amount for an additional fee). This resulted in P_1_ = 850 yen and P_2_ = 5,850 yen.

Statistical significance was defined as a two-sided *P*-value <0.05. All analyses were performed using Stata version 17.0, MP software (Stata Corp., College Station, TX, USA).

## RESULTS

### Participants characteristics

A total of 405,087 first-visit patients (mean age 54.9; standard deviation [SD], 20.2 years; 53.2% female) who visited hospitals with ≥200 beds in Ibaraki Prefecture were included in the analysis. Of these, 157,734 (mean age 60.3; SD, 16.0 years; 50.5% female; 38.9% of total first-visit patients) were referred. Table 1 shows the demographic characteristics of first-visit patients. Younger individuals were more likely to visit a hospital without a referral in both sexes. Among those ≥50 years, women were more likely to visit a hospital without a referral than men.

**Table 1.** Characteristics of first-visit patients with/without a referral

	First-visit patients with referral (*n* = 157,734)		First-visit patients without referral (*n* = 247,353)		Total (*n* = 405,087)	
		
	*n*	(%)	*n*	(%)	*n*	(%)
Age, years, mean (SD)	60.3	(16.0)	51.5	(21.9)	54.9	(20.2)
Age group, years						
0–9	2,881	(1.8)	18,693	(7.5)	21,574	(5.3)
10–19	4,047	(2.6)	14,514	(5.9)	18,561	(4.6)
20–29	4,246	(2.7)	14,438	(5.8)	18,684	(4.6)
30–39	6,980	(4.4)	18,868	(7.6)	25,848	(6.4)
40–49	11,618	(7.4)	23,852	(9.6)	35,470	(8.8)
50–59	15,032	(9.5)	23,795	(9.6)	38,827	(9.6)
60–69	60,593	(38.4)	79,023	(31.9)	139,616	(34.5)
70–74	52,337	(33.2)	54,170	(21.9)	106,507	(26.3)
Sex						
Female	79,595	(50.5)	135,891	(54.9)	215,486	(53.2)
Male	78,139	(49.5)	111,462	(45.1)	189,601	(46.8)
Female						
0–19 years	3,246	(4.1)	15,648	(11.5)	18,894	(8.8)
20–49 years	13,032	(16.4)	31,230	(23.0)	44,262	(20.5)
50–64 years	17,798	(22.4)	30,447	(22.4)	48,245	(22.4)
65–74 years	45,519	(57.2)	58,566	(43.1)	104,085	(48.3)
Male						
0–19 years	3,682	(4.7)	17,559	(15.8)	21,241	(11.2)
20–49 years	9,812	(12.6)	25,928	(23.3)	35,740	(18.9)
50–64 years	16,444	(21.0)	20,884	(18.7)	37,328	(19.7)
65–74 years	48,201	(61.7)	47,091	(42.2)	95,292	(50.3)
Hospital category						
Special Functioning Hospitals	22,116	(14.0)	8,824	(3.6)	30,940	(7.6)
Regional Medical Care Support Hospitals with ≥500 beds	20,359	(12.9)	17,760	(7.2)	38,119	(9.4)
Regional Medical Care Support Hospitals with 400–499 beds	55,704	(35.3)	69,374	(28.0)	125,078	(30.9)
Regional Medical Care Support Hospitals with 200–399 beds	45,341	(28.7)	68,762	(27.8)	114,103	(28.2)
Non-designated hospitals with ≥200 beds	14,214	(9.0)	82,633	(33.4)	96,847	(23.9)

SD, standard deviation.

### Crude referral rates before and after the mandate

Precisely 19 hospitals were included in the analysis: one SFH, two RMCSHs with ≥500 beds, five RMCSHs with 400–499 beds, seven RMCSHs with 200–399 beds, and four non-designated hospitals with ≥200 beds. Before the intervention period, the average crude referral rates were higher in the SFHs and the RMCSHs compared to non-designated hospitals (65.0% in the SFHs; 38.3%, 33.8%, and 34.8% in the RMCSHs with ≥500, 400–499, and 200–399 beds, respectively; and 11.7% in the non-designated hospitals). Referral rates increased across RMCSHs following the intervention, while the referral rate decreased in SFHs after the intervention (Table 2).

**Table 2.** Crude referral rates before and after the mandate according to hospital category

	Before mandate						After mandate					
	
	Number of first-visit patients		Number of referral patients		Referral rate (%)		Number of first-visit patients		Number of referral patients		Referral rate (%)	
					
	Mean	SD	Mean	SD	Mean	SD	Mean	SD	Mean	SD	Mean	SD
Special Functioning Hospitals	342.7	32.6	244.1	29.5	65.0	3.4	315.5	42.2	225.8	29.4	63.8	5.1
Regional Medical Care Support Hospitals with ≥500 beds	234.3	26.9	109.2	13.9	38.3	6.8	186.6	29.2	105.0	16.2	46.8	7.9
Regional Medical Care Support Hospitals with 400–499 beds	320.0	94.1	130.6	36.6	33.8	5.4	201.2	61.7	101.5	32.8	42.0	7.4
Regional Medical Care Support Hospitals with 200–399 beds	185.6	98.2	70.2	28.7	34.8	12.1	122.5	47.0	59.4	24.6	39.0	12.7
Non-designated hospitals with ≥200 beds	252.2	113.1	37.0	23.8	11.7	5.5	—	—	—	—	—	—

FY, fiscal year; SD, standard deviation.

Periods before/after the mandate are FY 2014–2015/FY 2016–2021 for Special Functioning Hospitals and Regional Medical Care Support Hospitals with ≥500 beds, FY 2014–2017/FY 2018–2021 for Regional Medical Care Support Hospitals with 400–499 beds, and FY 2014–2019/FY 2020–2021 for Regional Medical Care Support Hospitals with 200–399 beds.

The average numbers of patients per hospital are shown.

### Changes in referral rates: ITSA results

**Figure 1.**
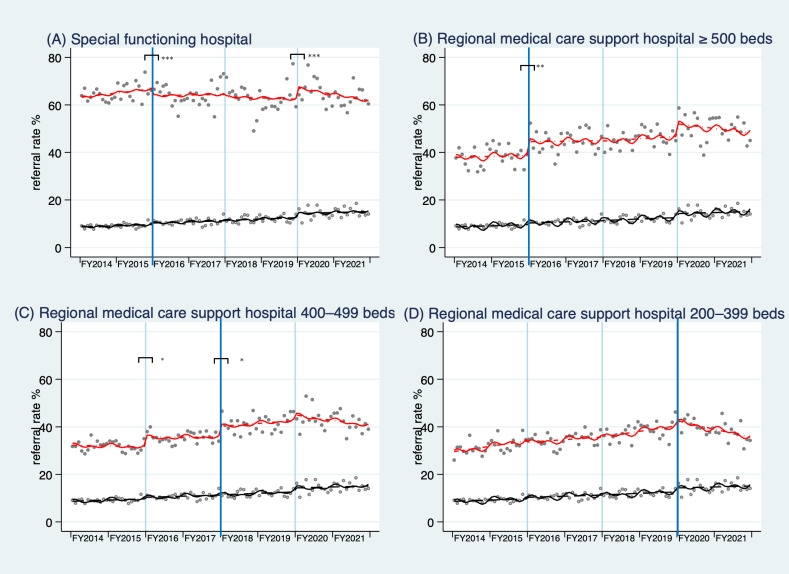
Trends in referral rates before and after the additional fee was mandated for non-referral first visits. The monthly referral rates are shown for (**A**) the Special Functioning Hospital (1 hospital), (**B**) Regional Medical Care Support Hospitals with ≥500 beds (2 hospitals), (**C**) Regional Medical Care Support Hospitals with 400–499 beds (5 hospitals), and (**D**) Regional Medical Care Support Hospitals with 200–399 beds (7 hospitals), compared to non-designated hospitals with ≥200 beds (4 hospitals, which serve as the control for all comparisons). The blue vertical line indicates the time when the corresponding hospitals are mandated to charge the additional fee. Bars with asterisks above the vertical lines indicate the statistical significance of the difference in level change between the treated and control groups: ^*^P < 0.05, ^**^P < 0.01, and ^***^P < 0.001. The average number of monthly first-visit patients in each category between April 2014 and March 2016 (period 1) is 342.7 in the Special Functioning Hospital, 468.6, 1797.0, 1674.3 in the Regional Medical Care Support Hospitals with ≥500, 400–499, and 200–399 beds, respectively, and 1544.8 in the non-designated hospitals with ≥200 beds. Circles represent the observed referral rates in the treated (closed circles) and control (open circles) groups. The solid line indicates the predicted referral rates of those treated in each category (red) and control (non-designated hospitals with ≥200 beds) (black). The dashed line depicts the deseasonalized trend in referral rates before and after the mandate for additional fees. FY, fiscal year.

Figure 1 and Table 3 present the results of the ITSA. The adjusted average referral rate to the SFHs estimated by ITSA was 54.7% (not shown in the tables or figures) before the intervention in April 2016. Following the intervention, the referral rate declined by 3.64 percentage points (95% confidence interval [CI], −4.60 to −2.68), with a −0.12 percentage point (95% CI, −0.22 to −0.02) decline in the trend thereafter. In contrast, among RMCSHs with ≥500 beds, the average referral rate increased by 5.10 percentage points (95% CI, 1.82–8.35) in April 2016 and remained stable. A similar trend was observed among RMCSHs with 400–499 beds, where the average referral rate increased by 3.54 percentage points (95% CI, 0.39–6.70) in April 2016, the timing when the additional fee was mandated for the SFHs and the RMCSHs with ≥500 beds, and remained stable thereafter. The referral rate further increased by 4.49 percentage points (95% CI, 0.28–8.70) in April 2018, when the additional fee was mandated for hospitals in this category. No significant changes were observed in referral rates among RMCSHs with 200–399 beds, while the referral rate for the SFHs was increased by 3.12 percentage points (95% CI, 2.31–3.93) at intervention 3 (April 2020).

**Table 3.** Changes in referral rates before and after the additional fee was mandated for non-referral first visits

	Intervention 1 (April 2016)				Intervention 2 (April 2018)				Intervention 3 (April 2020)			
		
	Level change^a^		Slope change^b^		Level change^a^		Slope change^b^		Level change^a^		Slope change^b^	
					
	Coef	95% CI	Coef	95% CI	Coef	95% CI	Coef	95% CI	Coef	95% CI	Coef	95% CI
SFH	**−3.64**	**(−4.60 to −2.68)**	**−0.12**	**(−0.22 to −0.02)**	−1.13	(−3.47 to 1.20)	−0.08	(−0.20 to 0.04)	**3.12**	**(2.31–3.93)**	**−0.18**	**(−0.26 to −0.09)**
RMCSH with ≥500 beds	**5.10**	**(1.84–8.35)**	−0.01	(−0.36 to 0.33)	−0.22	(−2.51 to 2.08)	0.05	(−0.07 to 0.17)	3.53	(−1.31 to 8.38)	−0.23	(0.37 to −0.08)
RMCSH with 400–499 beds	**3.54**	**(0.39–6.70)**	0.04	(−0.21 to 0.29)	**4.49**	**(0.28–8.70)**	0.09	(−0.10 to 0.28)	0.56	(−2.74 to 3.85)	−0.28	(−0.63 to 0.08)
RMCSH with 200–399 beds	−1.26	(−3.32 to 0.81)	−0.01	(−0.51 to 0.31)	0.06	(−3.64 to 3.75)	0.09	(−0.32 to 0.50)	0.23	(−2.01 to 2.47)	**−0.47**	**(−0.75 to −0.20)**

CI, confidence interval; Coef, coefficient; RMCSH, Regional Medical Care Support Hospital; SFH, Special Functioning Hospital.

^a^Difference in level change between treated and control.

^b^Difference in slope change between treated and control.

Special Functioning Hospitals and Regional Medical Care Support Hospitals with ≥500 beds are mandated in April 2016 (intervention 1), Regional Medical Care Support Hospitals with 400–499 beds are mandated in April 2018 (intervention 2), and Regional Medical Care Hospitals with 200–399 beds are mandated in April 2020 (intervention 3). Bold text indicates statistical significance.

### Price elasticity

The price elasticity was 0.04 for the SFHs and −0.08, −0.08, and 0.004 for the RMCSHs with ≥500 beds, 400–499 beds, and 200–399 beds, respectively.

## DISCUSSION

Using community-based health insurance claims data from a prefecture in Japan, we investigated changes in referral rates for first outpatient visits before and after the introduction of an additional fee, employing a controlled ITSA. The average referral rates increased when the additional fee was mandated among the RMCSHs with ≥400 beds.

The referral rate did not increase in SFHs after the intervention. However, the referral rate was relatively high (55% on average) even before the additional fee was introduced. This suggests that patients had already recognized the necessity of obtaining a referral for these hospitals (eg, university hospitals), likely influenced by policies implemented in tertiary care hospitals in other prefectures prior to the mandate. In contrast, referral rates in RMCSHs with ≥400 beds increased by 4–5 percentage points at the time of the intervention and remained stable thereafter, indicating that the mandate was effective among RMCSHs of this size. Additionally, the significant increase in referral rates for RMCSHs with 400–499 beds in April 2016—coinciding with the mandate for SFHs and RMCSHs with ≥500 beds—suggests a potential spillover effect.

There were no significant changes in the referral rates for RMCSHs with 200–399 beds. The coronavirus disease 2019 pandemic, which intensified in 2020, led to substantial changes in healthcare utilization due to factors such as reduced outpatient visits,^30^^,^^31^ postponed surgical procedures,^32^^,^^33^ and a decline in communicable diseases and trauma cases,^34^^,^^35^ especially during the first wave of the pandemic (April–June 2020 in Japan). As the intervention in April 2020 overlapped with this period, assessing its effectiveness was challenging. Further observation of referral rates in RMCSHs with 200–399 beds is needed.

Increasing the number of referrals may contribute to more efficient medical care by allocating medical resources. For patients, sharing information through referrals will reduce wasteful practices such as repeated examinations. Additionally, it is expected to reduce unnecessary medical expenditure. The effectiveness of medical resource allocation through financial incentives has been demonstrated through similar medical reforms. For example, China issued a Hierarchical Medical System in 2015 that included referral reform and financial incentives for patients (introducing different reimbursement ratios according to facility levels).^36^ This system has been associated with improved healthcare-seeking behaviors in urban areas^36^ and enhanced efficiency in medical resource allocation and the capacity of medical institutions.^37^ Similarly, tiered network plans in the United States categorize healthcare providers based on cost and quality, using differential cost-sharing mechanisms to encourage patients to seek care from preferred providers.^38^ Enrollment in such plans has been associated with a 5% reduction in total healthcare spending per member per quarter.^39^ Nevertheless, excessive restrictions on healthcare access could delay necessary care, potentially leading to increased hospitalizations or emergency visits. The price elasticity of RMCSHs with ≥400 beds, where referral rates increased statistically at the time of intervention, was similar to or less than that of previous studies. Specifically, the price elasticity of outpatient services in Japan ranged from −0.20 to −0.16 based on changes in the coinsurance rate at age 70 years.^11^^,^^12^ Among Medicare beneficiaries in the United States, the price elasticity of physician visits was −0.10, calculated based on changes in office visit copayments from $0 to $10.^10^ However, the study on Medicare beneficiaries also reported some potential offset effects, including a 5.4% increase in hospitalization expenditures, which was even greater among patients with multiple comorbidities.^10^ Moreover, the aforementioned Hierarchical Medical System in China could widen inequality in accessing healthcare resources among regions.^40^ Access to healthcare is often unintentionally limited, particularly in vulnerable populations. In-depth research is needed to evaluate whether healthcare outcomes, such as hospitalization or emergency visit rates, deteriorate after the introduction of additional fees. Furthermore, although we could not investigate the difference in price elasticities among income levels due to a lack of information, the differential effects of additional fees on patients having low income need to be clarified in future studies.

The survey on attitudes toward medical care in Japan reported that those who were older had a higher proportion of having a primary care physician (PCP).^41^ A lower proportion of those having a PCP may explain the lower referral rate in the younger population in both sexes. Although a higher percentage of women were reported to have PCPs than men, they are less likely to have a PCP specializing in internal medicine and more likely to have PCPs specializing in orthopedics, gynecology, ophthalmology, or other specialized fields.^41^ Consequently, women may directly visit higher-level hospitals without consulting other specialty PCPs about their internal manifestations. It is also essential to increase the proportion of PCPs specialized in general medicine to promote functional differentiation between clinics and higher-level hospitals.

This study had several limitations. First, the interventions were accompanied by revisions in medical fees. We cannot rule out the possibility that changes in other medical fees may have affected our results. However, each change in medical fees was small compared to additional fees, and the changes were applied equally to all healthcare facilities; we believe that the changes in medical fees do not affect patients’ choice of healthcare facilities. Second, we could not identify the first visit with a referral from the claims data because there was no referral code. Instead, we defined the first visit as a referral by extracting first-visit patients with claims for referral documents charged within 30 days before the first consultation. The number of participants and referral rates did not differ when we used different criteria (first-visit patients with claims of referral document charged within 60 days before the first consultation). However, we could not identify referral letters for a detailed examination after a health checkup from the claims data. The referral rates of RMCSHs should be at least 50% according to their approval requirements^42^; approximately 3–8% of referral rates might be underestimated due to this misclassification. Third, some patients are not charged an additional fee even if they visit without a referral, such as those who visit for a detailed examination after a health checkup, emergency patients (ie, those hospitalized on the same day), and those who have visited other hospital departments. We did not consider exemptions from the additional fee charges for these patients because we could not identify which patients for whom the additional fee had been waived. Fourth, some hospitals (at least one RMCSH with ≥500 beds and three RMCSHs with 200–399 beds) in the analysis began charging additional fees before they were mandated. Additionally, three non-designated hospitals also charged an additional fee of 1,000–2,000 yen. However, we believe that the conclusion does not change because this bias underestimates the association. Finally, as we used only data from the NHI in one prefecture, we cannot generalize the results nationwide. We did not include data from employment-based insurance programs covering full-time company employees, who are generally younger than the general population.

In conclusion, the average referral rate increased when the additional fee for non-referral was mandated among the RMCSHs with ≥400 beds. Further research is needed to evaluate whether the introduction of additional fees affects patient healthcare outcomes.

## SUPPLEMENTARY MATERIAL

eFigure 1. The expansion of mandated hospitals

eTable 1. Billing codes used for patient extraction

## References

[1] World Health Organization. World Report on Ageing and Health. https://iris.who.int/bitstream/handle/10665/186463/9789240694811_eng.pdf;jsessionid=CDF0AB3677039F2694029CA30DD3581A?sequence=1. Accessed 04.06.2025.

[2] Velasco Garrido M, Zentner A, Busse R. The effects of gatekeeping: a systematic review of the literature. Scand J Prim Health Care. 2011;29:28–38. 10.3109/02813432.2010.537015 21192758 PMC3347935

[3] Sripa P, Hayhoe B, Garg P, Majeed A, Greenfield G. Impact of GP gatekeeping on quality of care, and health outcomes, use, and expenditure: a systematic review. Br J Gen Pract. 2019;69:e294–e303. 10.3399/bjgp19X702209 30910875 PMC6478478

[4] Greenfield G, Foley K, Majeed A. Rethinking primary care’s gatekeeper role. BMJ. 2016;354:i4803. 10.1136/bmj.i4803 27662893

[5] Durand-Zaleski I. The French Health Care System. 01; 2019. https://www.commonwealthfund.org/international-health-policy-center/countries/france. Accessed 04.06.2025.

[6] Bíró A. Copayments, gatekeeping, and the utilization of outpatient public and private care at age 50 and above in Europe. Health Policy. 2013;111:24–33. 10.1016/j.healthpol.2013.03.009 23601570

[7] Dourgnon P, Naiditch M. The preferred doctor scheme: a political reading of a French experiment of gate-keeping. Health Policy. 2010;94:129–134. 10.1016/j.healthpol.2009.09.001 19819580

[8] Manning WG, Newhouse JP, Duan N, Keeler EB, Leibowitz A, Marquis MS. Health insurance and the demand for medical care: evidence from a randomized experiment. Am Econ Rev. 1987;77:251–277. 10284091

[9] Newhouse JP. *Free for All?: Lessons from the Rand Health Insurance Experiment*. Harvard University Press; 1993.

[10] Chandra A, Gruber J, McKnight R. Patient cost-sharing and hospitalization offsets in the elderly. Am Econ Rev. 2010;100:193–213. 10.1257/aer.100.1.193 21103385 PMC2982192

[11] Shigeoka H. The effect of patient cost sharing on utilization, health, and risk protection. Am Econ Rev. 2014;104:2152–2184. 10.1257/aer.104.7.2152

[12] Fukushima K, Mizuoka S, Yamamoto S, Iizuka T. Patient cost sharing and medical expenditures for the Elderly. J Health Econ. 2016;45:115–130. 10.1016/j.jhealeco.2015.10.005 26603160

[13] Miyawaki A, Noguchi H, Kobayashi Y. Impact of medical subsidy disqualification on children’s healthcare utilization: a difference-in-differences analysis from Japan. Soc Sci Med. 2017;191:89–98. 10.1016/j.socscimed.2017.09.001 28917140

[14] Kato D, Ryu H, Matsumoto T, . Building primary care in Japan: literature review. J Gen Fam Med. 2019;20:170–179. 10.1002/jgf2.252 31516802 PMC6732569

[15] Ministry of Health, Labour and Welfare. Patient’s Behavior Survey 2014 (Final Report). https://www.mhlw.go.jp/english/database/db-hss/pbs_2014.html; Accessed 24.07.2025.

[16] Medical care act; updated January 23, 2025. https://www.japaneselawtranslation.go.jp/en/laws/view/4006. Accessed 24.06.2025.

[17] Ikegami N, Yoo BK, Hashimoto H, . Japanese universal health coverage: evolution, achievements, and challenges. Lancet. 2011;378:1106–1115. 10.1016/S0140-6736(11)60828-3 21885107

[18] Ibaraki Prefectural Government. National health insurance situation; 2014 (in Japanese). https://www.pref.ibaraki.jp/hokenfukushi/koso/kokumin/koso/news/news-nhi-report2014.html; Accessed 24.07.2025.

[19] Ibaraki Prefectural Government. National health insurance situation; 2021 (in Japanese). https://www.pref.ibaraki.jp/hokenfukushi/koso/kokumin/koso/news/news-nhi-report2021.html; Accessed 24.07.2025.

[20] Japan Medical Association. Japan medical analysis platform. Ibaraki Prefcture (in Japanese); 2025. https://jmap.jp/cities/detail/pref/8. Accessed 04.06.2025.

[21] Ministry of Health, Labour and Welfare. Special Functioning Hospitals. https://www.mhlw.go.jp/english/wp/wp-hw3/dl/2-027.pdf. Accessed 04.06.2025.

[22] Ministry of Health, Labour and Welfare. Regional Medical Care Support Hospitals (from 1997). https://www.mhlw.go.jp/english/wp/wp-hw3/dl/2-028.pdf. Accessed 24.06.2025.

[23] Ministry of Health, Labour and Welfare. Overview of the Revision of Medical Fees in Fiscal; year 2016 (in Japanese). https://www.mhlw.go.jp/file/06-Seisakujouhou-12400000-Hokenkyoku/0000115977.pdf; Accessed 18.07.2025.

[24] Ministry of Health, Labour and Welfare. Overview of the Revision of Medical Fees in fiscal year; 2018 (in Japanese). https://www.mhlw.go.jp/file/06-Seisakujouhou-12400000-Hokenkyoku/0000198532.pdf; Accessed 24.07.2025.

[25] Ministry of Health, Labour and Welfare. Overview of the Revision of Medical Fees in fiscal year; 2020 (in Japanese). https://www.mhlw.go.jp/content/12400000/000605491.pdf; Accessed 24.07.2025.

[26] Shimizu S. (1) Nationwide list of insured medical institutions (hospitals and clinics), (2) Nationwide list of health care pharmacies, (3) Correspondence table of postal codes and secondary medical districts. Mon IHEP. 2015;241:22–25.

[27] Wagner AK, Soumerai SB, Zhang F, Ross-Degnan D. Segmented regression analysis of interrupted time series studies in medication use research. J Clin Pharm Ther. 2002;27:299–309. 10.1046/j.1365-2710.2002.00430.x 12174032

[28] Bernal JL, Cummins S, Gasparrini A. Interrupted time series regression for the evaluation of public health interventions: a tutorial. Int J Epidemiol. 2017;46:348–355. 10.1093/ije/dyw098 27283160 PMC5407170

[29] Keeler EB, Rolph JE. The demand for episodes of treatment in the health insurance experiment. J Health Econ. 1988;7:337–367. 10.1016/0167-6296(88)90020-3 10312839

[30] Ikesu R, Miyawaki A, Sugiyama T, Nakamura M, Ninomiya H, Kobayashi Y. Trends in diabetes care during the COVID-19 outbreak in Japan: an observational study. J Gen Intern Med. 2021;36:1460–1462. 10.1007/s11606-020-06413-w 33469742 PMC7814982

[31] Morishita T, Takada D, Shin JH, . Effects of the COVID-19 pandemic on heart failure hospitalizations in Japan: interrupted time series analysis. ESC Heart Fail. 2022;9:31–38. 10.1002/ehf2.13744 34913269 PMC8788142

[32] Okuno T, Takada D, Shin JH, . Surgical volume reduction and the announcement of triage during the 1st wave of the COVID-19 pandemic in Japan: a cohort study using an interrupted time series analysis. Surg Today. 2021;51:1843–1850. 10.1007/s00595-021-02286-6 33881619 PMC8059122

[33] Miyawaki A, Tomio J, Nakamura M, Ninomiya H, Kobayashi Y. Changes in surgeries and therapeutic procedures during the COVID-19 outbreak: a longitudinal study of acute care hospitals in Japan. Ann Surg. 2021;273:e132–e134. 10.1097/SLA.0000000000004528 33214438 PMC7959863

[34] Sano K, Nakamura M, Ninomiya H, Kobayashi Y, Miyawaki A. Large decrease in paediatric hospitalisations during the COVID-19 outbreak in Japan. BMJ Paediatr Open. 2021;5:e001013. 10.1136/bmjpo-2020-001013 34192195 PMC7956728

[35] Nagano H, Takada D, Shin JH, Morishita T, Kunisawa S, Imanaka Y. Hospitalization of mild cases of community-acquired pneumonia decreased more than severe cases during the COVID-19 pandemic. Int J Infect Dis. 2021;106:323–328. 10.1016/j.ijid.2021.03.074 33794382 PMC8006513

[36] Zhou Z, Zhao Y, Shen C, Lai S, Nawaz R, Gao J. Evaluating the effect of hierarchical medical system on health seeking behavior: a difference-in-differences analysis in China. Soc Sci Med. 2021;268:113372. 10.1016/j.socscimed.2020.113372 32979776

[37] Liang C, Zhao Y, Yu C, Sang P, Yang L. Hierarchical medical system and local medical performance: a quasi-natural experiment evaluation in Shanghai, China. Front Public Health. 2022;10:904384. 10.3389/fpubh.2022.904384 36324471 PMC9619052

[38] Frank MB, Hsu J, Landrum MB, Chernew ME. The impact of a tiered network on hospital choice. Health Serv Res. 2015;50:1628–1648. 10.1111/1475-6773.12291 25752219 PMC4600365

[39] Sinaiko AD, Landrum MB, Chernew ME. Enrollment in a health plan with a tiered provider network decreased medical spending by 5 percent. Health Aff (Millwood). 2017;36:870–875. 10.1377/hlthaff.2016.1087 28461354

[40] Lu C, Zhang Z, Lan X. Impact of China’s referral reform on the equity and spatial accessibility of healthcare resources: a case study of Beijing. Soc Sci Med. 2019;235:112386. 10.1016/j.socscimed.2019.112386 31272079

[41] Japan Medical Association Research Institute. The survey on attitudes toward medical care in Japan 6^th^. https://www.jmari.med.or.jp/download/WP384_data.pdf. Accessed 25.08.2025.

[42] Ministry of Health, Labour and Welfare. About Regional Medical Care Support Hospitals. https://www.mhlw.go.jp/stf/seisakunitsuite/bunya/0000137801_00015.html. Accessed 18.08.2025.

