# Supplementary material for: Changes in Referral Rates After the Mandate of Charging Additional Fees for Non-referral First Visits: A Controlled Interrupted Time-series Analysis
Source: J Epidemiol. 2026 May 5;36(5):155–61. doi: 10.2188/jea.JE20250285 (PMC13085651; doi:10.2188/jea.JE20250285)
Supplement: Supplementary file 1 [file je-36-155-s001.pdf]

eFigure 1. The expansion of mandated hospitals

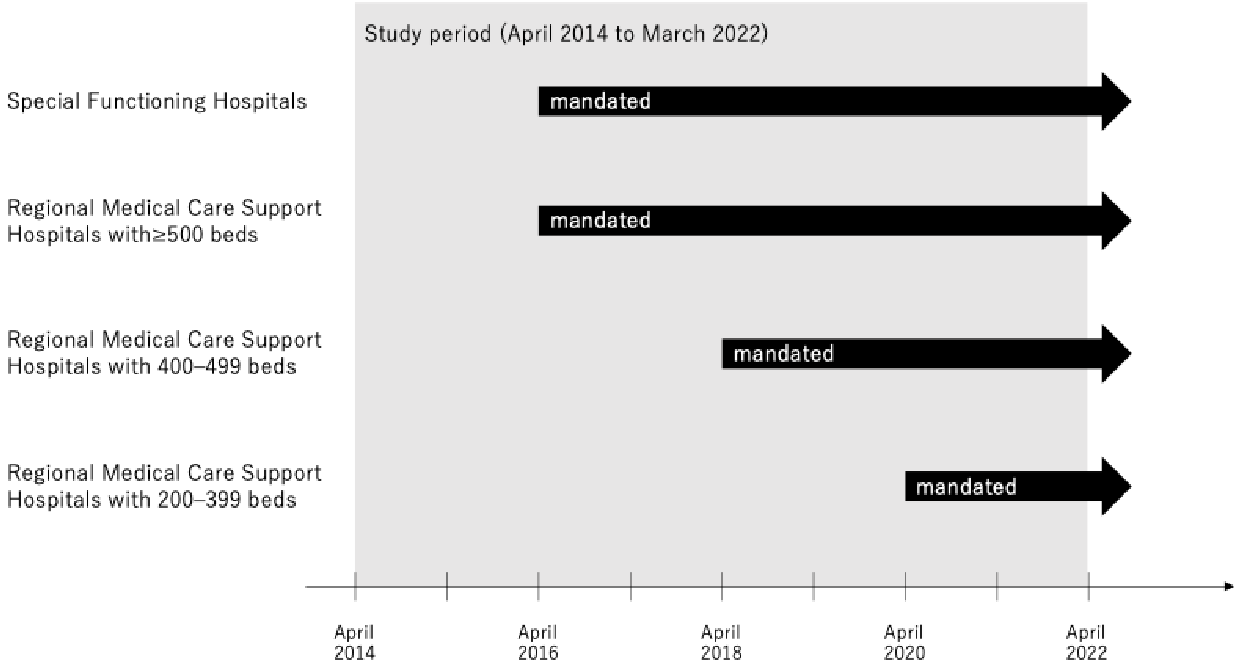

eTable 1. Billing codes used for patient extraction

	Billing codes
First consultation fee	Medical practice with Japanese medical procedure code of first consultation fee (111000110)
Emergency department visits	Medical practice under the following Japanese medical procedure codes:<br>– Out of hours surcharge (111000570, 111000870, 111011970, 111012270)<br>– Holiday surcharge (111000670, 111012070, 111011670)<br>– Late night surcharge (111000770, 111012170, 111011770)<br>– Early morning surcharge (111012470, 11011570)<br>– Regional liaison night/holiday consultation fee (113011610)<br>– Regional liaison night/holiday pediatric consultation fee (113006710, 113008210)<br>– In-hospital triage management fee (113013710)<br>– Night/holiday emergency transport medical management fee (113013810)<br>– Medical equipment safety management fee (113011210, 113011310)
Emergency transport	Medical practice with Japanese procedure code of night/holiday emergency transport medical management fee (113013810)
Referral document fee	Medical practice with Japanese medical procedure code of referral document fee (180016110, 113009510)
